# Decreased Incidence of Infections Caused by Pathogens Transmitted Commonly Through Food During the COVID-19 Pandemic — Foodborne Diseases Active Surveillance Network, 10 U.S. Sites, 2017–2020

**DOI:** 10.15585/mmwr.mm7038a4

**Published:** 2021-09-24

**Authors:** Logan C. Ray, Jennifer P. Collins, Patricia M. Griffin, Hazel J. Shah, Michelle M. Boyle, Paul R. Cieslak, John Dunn, Sarah Lathrop, Suzanne McGuire, Tamara Rissman, Elaine J Scallan Walter, Kirk Smith, Melissa Tobin-D’Angelo, Katie Wymore, Joanna Zablotsky Kufel, Beverly J. Wolpert, Robert Tauxe, Daniel C. Payne

**Affiliations:** ^1^Division of Foodborne, Waterborne, and Environmental Diseases, National Center for Emerging and Zoonotic Infectious Diseases, CDC; ^2^Maryland Department of Health; ^3^Oregon Health Authority; ^4^Tennessee Department of Health; ^5^University of New Mexico, Albuquerque, New Mexico; ^6^New York State Department of Health; ^7^Connecticut Emerging Infections Program, New Haven, Connecticut; ^8^Colorado School of Public Health, University of Colorado, Anschutz Medical Campus, Aurora, Colorado; ^9^Minnesota Department of Health; ^10^Georgia Department of Public Health; ^11^California Emerging Infections Program, Oakland, California; ^12^Food Safety and Inspection Service, U.S. Department of Agriculture, Washington, DC; ^13^Center for Food Safety and Applied Nutrition, Food and Drug Administration, Silver Spring, Maryland

Foodborne illnesses are a substantial and largely preventable public health problem; before 2020 the incidence of most infections transmitted commonly through food had not declined for many years. To evaluate progress toward prevention of foodborne illnesses in the United States, the Foodborne Diseases Active Surveillance Network (FoodNet) of CDC’s Emerging Infections Program monitors the incidence of laboratory-diagnosed infections caused by eight pathogens transmitted commonly through food reported by 10 U.S. sites.* FoodNet is a collaboration among CDC, 10 state health departments, the U.S. Department of Agriculture’s Food Safety and Inspection Service (USDA-FSIS), and the Food and Drug Administration. This report summarizes preliminary 2020 data and describes changes in incidence with those during 2017–2019. During 2020, observed incidences of infections caused by enteric pathogens decreased 26% compared with 2017–2019; infections associated with international travel decreased markedly. The extent to which these reductions reflect actual decreases in illness or decreases in case detection is unknown. On March 13, 2020, the United States declared a national emergency in response to the COVID-19 pandemic. After the declaration, state and local officials implemented stay-at-home orders, restaurant closures, school and child care center closures, and other public health interventions to slow the spread of SARS-CoV-2, the virus that causes COVID-19 (*1*). Federal travel restrictions were declared (*1*). These widespread interventions as well as other changes to daily life and hygiene behaviors, including increased handwashing, have likely changed exposures to foodborne pathogens. Other factors, such as changes in health care delivery, health care–seeking behaviors, and laboratory testing practices, might have decreased the detection of enteric infections. As the pandemic continues, surveillance of illness combined with data from other sources might help to elucidate the factors that led to the large changes in 2020; this understanding could lead to improved strategies to prevent illness. To reduce the incidence of these infections concerted efforts are needed, from farm to processing plant to restaurants and homes. Consumers can reduce their risk of foodborne illness by following safe food-handling and preparation recommendations.

FoodNet conducts active, population-based surveillance of laboratory-diagnosed infections caused by *Campylobacter, Cyclospora, Listeria, Salmonella,* Shiga toxin-producing *Escherichia coli* (STEC), *Shigella, Vibrio,* and *Yersinia* reported from 10 sites covering approximately 15% of the U.S. population (approximately 50 million persons per U.S. Census Bureau estimates in 2019). Bacterial infections are defined as isolation of bacteria from a clinical specimen by culture or detection of pathogen antigen, nucleic acid sequence, or, for STEC,^†^ Shiga toxin or Shiga toxin genes by a culture-independent diagnostic test (CIDT).^§^ *Listeria* infections are defined as isolation of *L. monocytogenes* or detection of its nucleic acid sequences from a normally sterile site, or from placental or fetal tissue in the instance of miscarriage or stillbirth. *Cyclospora* infections are defined as detection of the parasite using ultraviolet fluorescence microscopy, specific stains, or polymerase chain reaction. 

In this analysis, patients with no history of international travel or unknown travel were considered to have domestically acquired infection.^¶^ Death was attributed to infection when it occurred during hospitalization or within 7 days after specimen collection for nonhospitalized patients. Incidence (cases per 100,000 population) was calculated by dividing the number of infections in 2020 by the U.S. Census estimates of the surveillance area population for 2019. Incidence measures included all laboratory-diagnosed infections. A negative binomial model with 95% confidence intervals (CIs) was used to estimate change in incidence during 2020 compared with those during 2017–2019, adjusting for changes in the population over time.

Surveillance for physician-diagnosed post-diarrheal hemolytic uremic syndrome (HUS), a complication of STEC infection characterized by renal failure, thrombocytopenia, and microangiopathic anemia, was conducted through a network of nephrologists and infection preventionists and by hospital discharge data review. This report includes HUS data for children aged <18 years for 2019, the most recent year for which data are available. FoodNet surveillance activities were reviewed by CDC and were conducted consistent with applicable federal law and CDC policy.**

During 2020, FoodNet identified 18,462 cases of infection, 4,788 hospitalizations, and 118 deaths (Table). The overall incidence was highest for *Campylobacter* (14.4 per 100,000 population), followed by *Salmonella* (13.3), STEC (3.6), *Shigella* (3.1), *Yersinia* (0.9), *Vibrio* (0.7), *Cyclospora* (0.6), and *Listeria* (0.2). During 2020, 26% fewer infections were reported compared with the average annual number reported during 2017–2019; the incidence in 2020 was significantly lower for all pathogens except *Yersinia* and *Cyclospora*. The percentage of infections resulting in hospitalization increased 2% compared with 2017–2019 (Figure 1). During 2020, 5% (958) of infections were associated with international travel compared with 14% during 2017–2019. In 2020, most (798; 83%) of these infections occurred during January–March. 

**TABLE:** Number of laboratory-diagnosed bacterial and parasitic infections, hospitalizations, deaths, and outbreak-associated cases, incidence, and percentage change compared with 2017–2019 average annual incidence, by pathogen — Foodborne Diseases Active Surveillance Network, 10 U.S. sites,* 2017–2020^†^

Pathogens	No. of infections^§^	No. (%)			Incidence^§§^	% change in incidence, 2017–2019 to 2020 (95%CI)^¶¶^
		Hospitalizations^¶^	Deaths**	Outbreak-associated cases^††^		
**Bacteria**						
*Campylobacter*	7,208	1,524 (21)	25 (0.3)	19 (0.3)	14.4	−23 (−29 to −16)
*Salmonella*	6,694	1,971 (29)	48 (0.7)	631 (9)	13.3	−22 (−26 to −17)
STEC***	1,824	441 (24)	7 (0.4)	27 (1)	3.6	−37 (−47 to −26)
*Shigella*	1,534	524 (34)	3 (0.2)	145 (9)	3.1	−41 (−54 to −23)
*Yersinia*	455	119 (26)	5 (1.1)	0 (—)	0.9	−10 (−29 to 14)
*Vibrio*	330	88 (27)	8 (2.4)	0 (—)	0.7	−25 (−39 to −8)
*Listeria*	104	99 (95)	22 (21.2)	2 (2)	0.2	−27 (−43 to −7)
**Parasite**						
*Cyclospora*	313	22 (7)	0 (—)	116 (37)	0.6	−17 (−50 to 37)
**Total**	**18,462**	**4,788 (26)**	**118 (0.6)**	**940 (5)**	**N/A**	**N/A**

**Abbreviations:** CI = confidence interval; CIDT = culture-independent diagnostic test; N/A = not applicable; STEC = Shiga toxin-producing *Escherichia coli*.

* Data were obtained from Connecticut, Georgia, Maryland, Minnesota, New Mexico, Oregon, Tennessee, and selected counties in California, Colorado, and New York.

^†^ Data for 2020 are preliminary.

^§^ Bacterial infections were diagnosed by positive CIDT or isolation by culture, or for STEC by detection of Shiga toxin or Shiga toxin genes. *Cyclospora* infections were diagnosed by detection of parasite by ultraviolet fluorescence microscopy or polymerase chain reaction.

^¶^ Defined as hospitalizations occurring within 7 days before or after specimen collection and emergency department stays of >24 hours during the same time frame. Change in percentage of infections resulting in hospitalization during 2020 compared with 2017–2019: *Campylobacter* (+1), *Salmonella* (+2), STEC (+2), *Shigella* (+8), *Yersinia* (+3), *Vibrio* (−3), *Listeria* (−1), *Cyclospora* (+2).

** Defined as deaths occurring during hospitalization or within 7 days after specimen collection for persons who were not hospitalized. Change in percentage of infections resulting in death during 2020 compared with 2017–2019: *Campylobacter* (0.0), *Salmonella* (+0.2), STEC (0.0), *Shigella* (+0.1), *Yersinia* (+0.2), *Vibrio* (0.0), *Listeria* (+2.0), *Cyclospora* (−0.1).

^††^ Change in percentage of infections associated with outbreaks during 2020 compared with 2017–2019: *Campylobacter* (−0.1), *Salmonella* (+2), STEC (−4), *Shigella* (+6), *Yersinia* (0.0), *Vibrio* (−4), *Listeria* (−3), *Cyclospora* (+8).

^§§^ Per 100,000 population.

^¶¶^ Percentage change reported as increase or decrease.

*** The incidence of STEC O157 infections (0.5 per 100,000 population) changed by −37% (95% CI = −49 to −22) compared with 2017–2019; the incidence of non-O157 STEC infections (1.4) changed by −43% (95% CI = −51 to −34).

**FIGURE 1:**
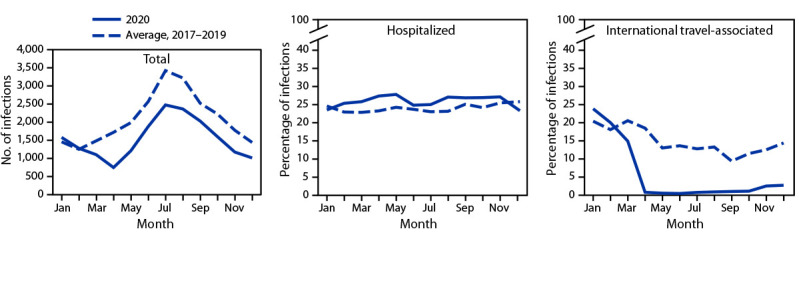
Number of laboratory-diagnosed bacterial and parasitic infections, percentage of patients hospitalized,* and percentage with international travel,^†^ by month — Foodborne Diseases Active Surveillance Network, 10 U.S. sites,^§^ 2017–2020^¶^ * Hospital admission in the 7 days before or after specimen collection among those with known information; it was unknown for 8% of infections during 2020 and 4% during 2017–2019. †History of international travel in the 30 days before illness began for *Listeria* and *Salmonella* serotypes Typhi and Paratyphi, 15 days before illness began for *Cyclospora*, and 7 days before illness began for other pathogens. International travel was unknown for 26% of infections during 2020 and 17% during 2017–2019. During 2020, 5% (958) of infections were associated with international travel compared with 14% during 2017–2019. In 2020, most (798; 83%) of these infections occurred during January–March. ^§^ Data were obtained from Connecticut, Georgia, Maryland, Minnesota, New Mexico, Oregon, Tennessee, and selected counties in California, Colorado, and New York. ^¶^ Data for 2020 are preliminary.

Overall, 59% of bacterial infections were diagnosed using a CIDT (range = 14% [*Listeria*] to 100% [STEC]) (Figure 2); this was a 2% increase from 2017−2019. The percentage diagnosed using only a CIDT (i.e., including specimens with negative cultures and those not cultured) was 1% higher during 2020 than the percentage during 2017−2019. Among specimens with a positive CIDT result during 2020, a reflex culture^††^ was performed for 73%, which was 2% lower than during 2017–2019. Reflex cultures decreased for *Vibrio* (by 15%), *Yersinia* (7%), *Campylobacter* (5%), and STEC (2%); increased for *Salmonella* (2%), and *Shigella* (2%); and did not change for *Listeria*.

**FIGURE 2:**
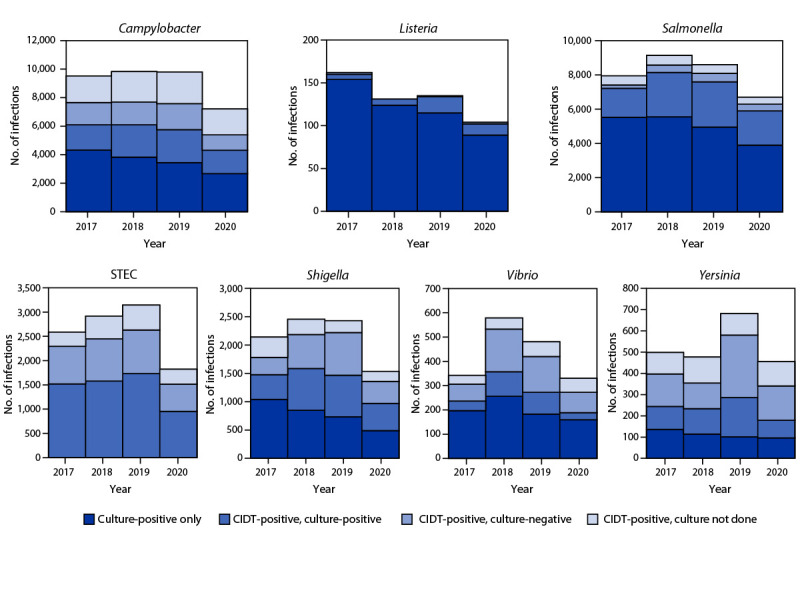
Number of infections diagnosed by culture or culture-independent diagnostic test, by pathogen, year, and culture status — Foodborne Diseases Active Surveillance Network, 10 U.S. sites,* 2017–2020^†^ **Abbreviations: **CIDT = culture-independent diagnostic test; STEC = Shiga toxin-producing *Escherichia coli*. * Data were obtained from Connecticut, Georgia, Maryland, Minnesota, New Mexico, Oregon, Tennessee, and selected counties in California, Colorado, and New York. † Data for 2020 are preliminary.

Among 5,336 (91%) fully serotyped *Salmonella* isolates in 2020, the seven most common serotypes were Enteritidis (1.6 per 100,000 population), Newport (1.5), Javiana (1.0), Typhimurium (0.9), I 4,[5],12:i:- (0.5), Hadar (0.4), and Infantis (0.3). Compared with 2017–2019, incidence during 2020 was significantly lower for I 4,[5],12:i:- (48% lower), Typhimurium (37% lower), Enteritidis (36% lower), and Javiana (31% lower). Incidence was significantly higher for Hadar (617% higher; 95% CI = 382–967) and did not change significantly for Newport or Infantis. Most (73%) of the 631 outbreak-associated *Salmonella* infections during 2020 were caused by three serotypes: Newport (220; 35%), Hadar (135; 21%), and Enteritidis (108; 17%). All outbreak-associated Hadar infections were from one multistate outbreak linked to contact with backyard poultry; 47 (35%) illnesses resulted in hospitalization. Four serogroups accounted for 63% of the 955 culture-positive STEC isolates. Serogroup O157 was most common (264; 28%), followed by O26 (148; 15%), O103 (115; 12%), and O111 (78; 8%).

FoodNet identified 63 cases of post-diarrheal HUS in children aged <18 years (0.6 cases per 100,000 population) during 2019; 55 (87%) had evidence of STEC infection and 41 (65%) were in children aged <5 years (1.4 per 100,000 population). These rates were similar to those during 2016–2018.

## Discussion

The 26% decrease in incidence of infections caused by pathogens transmitted commonly through food during 2020 was the largest single-year variation in incidence during 25 years of FoodNet surveillance; widespread public health interventions implemented to prevent SARS-CoV-2 transmission might have contributed to this decrease. For example, infections associated with international travel decreased markedly after pandemic-related travel restrictions were imposed. Other interventions, such as restaurant closures, might have contributed to declines in incidence. However, a higher than usual proportion of infections might have been undetected because factors such as changes in health care-seeking behaviors, and broader use of telehealth might have limited the number of stool specimens tested. Marked decreases in emergency department visits for abdominal pain and other digestive or abdominal signs and symptoms occurred early in the pandemic (*2*). The proportion of infections resulting in hospitalization increased slightly; possible explanations include disproportionate decreases in health care-seeking among those with milder illness or delayed health care-seeking resulting in more severe illness at the time of clinical presentation.

The proportion of infections diagnosed by culture versus CIDTs was stable during 2020, suggesting that a change in clinical laboratory testing practices was not a major contributor to the decreased incidence of infections. Before 2020, the incidence of *Campylobacter*, *Salmonella*, STEC, *Vibrio*, *Yersinia*, and *Cyclospora* infections had been increasing; the addition of infections diagnosed by CIDTs to FoodNet surveillance beginning in 2012, and the introduction of DNA-based syndrome panels^§§^ in 2016 likely contributed to the increases (*3*).

Changes in clinical and public health laboratory capacity in response to the COVID-19 pandemic might have contributed to observed decreases in reflex culturing. Before 2020, reflex culture of specimens positive for *Campylobacter*, *Salmonella*, *Shigella*, and *Yersinia* increased in FoodNet sites, augmented by CDC funding. Until metagenomic CIDTs are developed, culture is necessary to identify pathogen subtypes, antimicrobial resistance patterns, and whole-genome sequences (*4*). Fewer cultures decrease the ability to detect and investigate outbreaks and sporadic cases of emerging pathogens, which relies on sequencing.

The incidences of *Salmonella* Infantis, *Cyclospora,* and *Yersinia* infections, which had previously been increasing, did not change, possibly because of continuing prepandemic factors that led to rising incidences during previous years (*5*); the stable incidences despite the pandemic suggest that they might have increased otherwise. As pandemic-related restrictions are lifted, illnesses caused by these pathogens and by Hadar, the one *Salmonella* serotype with increasing incidence, should be closely monitored. Rising multidrug resistant *Salmonella* Infantis infections have been linked to consumption of chicken (*6*–*8*). Hadar infections have been linked to backyard flocks and to consumption of turkey (*8*,*9*). USDA-FSIS did not detect a significantly higher percentage of *Salmonella* Hadar in raw poultry samples collected in 2020 compared with 2017–2019 (USDA-FSIS, unpublished data, 2021). Typhimurium continued to decline in rank among *Salmonella* serotypes, dropping to fourth most common for the first time.

The findings in this report are subject to at least three limitations. First, the pandemic and corresponding public health response make explaining changes in the observed incidences of infections challenging. Second, changes in health care-seeking behaviors and health care delivery during the pandemic likely limited ascertainment of cases. Finally, sites reported decreases that varied over time in the willingness of ill persons to be interviewed and in staff member capacity to conduct case interviews; these factors might have resulted in missing data and recall bias.

Public health interventions to prevent SARS-CoV-2 transmission likely influenced exposures associated with enteric diseases, resulting in real declines in incidence, as evidenced by decreased numbers of infections associated with international travel. Continued surveillance might improve the understanding of how the pandemic affected foodborne illness and might help identify prevention measures and strategies that target particular pathogens and foods. To reduce the incidence of these infections concerted efforts are needed, from farm to processing plant to restaurants and homes. Consumers can reduce their risk of foodborne illness by following safe food-handling and preparation recommendations.

**Summary**

**What is already known about this topic?**

Before 2020, the incidence of infections transmitted commonly by food had not declined for many years.

**What is added by this report?**

During 2020, FoodNet identified 26% fewer infections compared with the average annual number during 2017–2019, including decreased infections associated with international travel.

**What are the implications for public health practice?**

The pandemic and resulting public health response present challenges to explaining changes in observed foodborne illness incidences. Continued surveillance might help elucidate the impact of the COVID-19 pandemic on foodborne illness and identify strategies to decrease illnesses. Concerted efforts are needed to reduce the incidence of these infections from farm to processing plant to restaurants and homes. Consumers can reduce their risk of foodborne illness by following safe food-handling and preparation recommendations.

## Footnotes

* Data were obtained from Connecticut, Georgia, Maryland, Minnesota, New Mexico, Oregon, Tennessee, and selected counties in California, Colorado, and New York (https://www.cdc.gov/foodnet).

† STEC infections are defined as identification of Shiga toxin or its genes by any laboratory.

§ A CIDT detects the presence of a specific antibody or antigen or the DNA of an organism.

¶ History of international travel in the 30 days before illness began for *Listeria *and *Salmonella *serotypes Typhi and Paratyphi, 15 days before illness began for *Cyclospora*, and 7 days before illness began for other pathogens.

** 45 C.F.R. part 46.102(l)(2), 21 C.F.R. part 56; 42 U.S.C. Sect. 241(d); 5 U.S.C. Sect. 552a; 44 U.S.C. Sect. 3501 et seq.

†† Culture of a specimen with a positive CIDT result.

§§ Syndromic panels are commercial CIDTs that simultaneously detect multiple pathogens associated with clinical syndromes, such as diarrheal illness.

## References

[1] Schuchat A; CDC COVID-19 Response Team. Public health response to the initiation and spread of pandemic COVID-19 in the United States, February 24–April 21, 2020. MMWR Morb Mortal Wkly Rep 2020;69:551–6. 10.15585/mmwr.mm6918e2 32379733 PMC7737947

[2] Hartnett KP, Kite-Powell A, DeVies J, ; National Syndromic Surveillance Program Community of Practice. Impact of the COVID-19 pandemic on emergency department visits—United States, January 1, 2019–May 30, 2020. MMWR Morb Mortal Wkly Rep 2020;69:699–704. 10.15585/mmwr.mm6923e1 32525856 PMC7315789

[R3] Marder EP, Griffin PM, Cieslak PR, Preliminary incidence and trends of infections with pathogens transmitted commonly through food—Foodborne Diseases Active Surveillance Network, 10 U.S. sites, 2006–2017. MMWR Morb Mortal Wkly Rep 2018;67:324–8. 10.15585/mmwr.mm6711a3 29565841 PMC5868202

[4] Carleton HA, Besser J, Williams-Newkirk AJ, Huang A, Trees E, Gerner-Smidt P. Metagenomic approaches for public health surveillance of foodborne infections: opportunities and challenges. Foodborne Pathog Dis 2019;16:474–9. 10.1089/fpd.2019.2636 31170005 PMC6653786

[5] Tack DM, Ray L, Griffin PM, Preliminary incidence and trends of infections with pathogens transmitted commonly through food—Foodborne Diseases Active Surveillance Network, 10 U.S. Sites, 2016–2019. MMWR Morb Mortal Wkly Rep 2020;69:509–14. 10.15585/mmwr.mm6917a1 32352955 PMC7206985

[6] The National Antimicrobial Resistance Monitoring System. NARMS integrated report, 2016–2017. Laurel, MD: US Department of Health and Human Services, Food and Drug Administration; 2019. https://www.fda.gov/animal-veterinary/national-antimicrobial-resistance-monitoring-system/2016-2017-narms-integrated-summary

[7] Tyson GH, Li C, Harrison LB, A multidrug-resistant *Salmonella* Infantis clone is spreading and recombining in the United States. Microb Drug Resist 2021;27:792–9. 10.1089/mdr.2020.0389 33232624 PMC11555764

[8] Jackson BR, Griffin PM, Cole D, Walsh KA, Chai SJ. Outbreak-associated *Salmonella enterica* serotypes and food commodities, United States, 1998-2008. Emerg Infect Dis 2013;19:1239–44. 10.3201/eid1908.121511 23876503 PMC3739514

[9] CDC. Outbreaks of *Salmonella* infections linked to backyard poultry—United States, January–November 2020. Atlanta, GA: US Department of Health and Human Services, CDC; 2020. https://www.cdc.gov/salmonella/backyardpoultry-05-20/index.html

